# An amplicon-based tiled PCR scheme for the enrichment of avian metapneumovirus subtype B genomes prior to next generation sequencing

**DOI:** 10.3389/fcimb.2026.1782197

**Published:** 2026-03-17

**Authors:** Sonsiray Álvarez-Narváez, David L. Suarez, Darrell R. Kapczynski

**Affiliations:** U.S. National Poultry Research Center, United States Department of Agriculture, Agricultural Research Service, Athens, GA, United States

**Keywords:** amplicon-based tiled PCR, avian metapneumovirus B, genome enrichment, long-read sequencing, next generation sequencing, short-read sequencing

## Abstract

Avian metapneumovirus (aMPV) is an important respiratory pathogen of global concern in poultry. Until recently, the United States (U.S.) was considered free of aMPV following the eradication of subtype C in the early 2000s. However, in 2023–2024, both aMPV subtypes A and B were confirmed in the U.S., leading to widespread outbreaks in more than 30 states. The rapid spread of these viruses, despite enhanced biosecurity measures, highlights critical gaps in our epidemiological understanding and underscores the urgent need for active surveillance programs. Whole genome sequencing has become a valuable tool for the characterization and epidemiological investigation of viruses, and viral genome enrichment methods are essential to increase the proportion of viral genetic materials in a sample prior sequencing. In this study, we evaluated two aMPV subtype B genome enrichment strategies: the currently used combination of host and bacterial RNA depletion followed by sequence-independent single-primer amplification (HD-SISPA), and a newly developed amplicon-based tiled PCR approach (aMPVB-ATP). Samples treated with aMPVB-ATP yielded significantly higher proportion of aMPV reads (41%) following short-read next-generation sequencing (NGS) compared to those treated with HD-SISPA (1%). This improvement enabled the recovery of complete or nearly complete aMPV genomes from samples with RT-PCR cycle threshold (Ct) values below 22. The compatibility of aMPVB-ATP with long-read NGS was also assessed, showing no significant differences in genome coverage or sequencing depth compared to its performance combined with short-read platforms.

## 1 Introduction

Avian metapneumovirus (aMPV) are single-stranded, non-segmented negative-sense RNA viruses, belonging to the *Metapneumovirus* genus within the *Pneumoviridae* family (Rima et al., 2017). aMPV are major respiratory pathogens in poultry, causing outbreaks in turkeys and chickens worldwide (Salles et al., 2023). While domestic poultry are considered the primary hosts (Brown et al., 2019), aMPV have also been isolated from wild bird species, including ducks, geese, gulls, and pheasants (Turpin et al., 2008; Graziosi et al., 2022). Genetically, aMPVs are divided into four established subtypes: aMPV -A, -B, -C, and -D (Bäyon-Auboyer et al., 2000). More recently, three novel and genetically divergent aMPV strains, GuMPV B25, YS24 and PAR-05, have been identified in wild avian hosts (Canuti et al., 2019; Retallack et al., 2019).

Following the eradication of aMPV-C in the early 2000s (Franzo et al., 2024; Goraichuk et al., 2024c; Luqman et al., 2024), the United States (U.S.) had remained free of aMPV until the re-emergence of subtypes A and B in late 2023 and early 2024. The first cases were reported in California (aMPV-A) and North Carolina (aMPV-B) during December 2023, and in only eight months, by August 2024, aMPV-A and -B had spread to 28 states. Currently, confirmed cases of aMPV-A and -B had been reported in chicken and turkey flocks across 32 states, resulting in high economic losses (Klipstein, 2025; Zimmerman, 2025). aMPV had rapid dissemination throughout U.S. poultry operations (Luqman et al., 2024) despite the implementation of strict biosecurity protocols following the 2015 highly pathogenic avian influenza outbreak (Animal and Plant Health Inspection Service U.S, 2024), points to critical deficiencies in current epidemiological insight, and emphasizes the pressing need for robust active surveillance strategies.

Whole genome sequencing (WGS) has become an important tool for studying the molecular epidemiology of many viruses, including aMPV (Goraichuk et al., 2024c; Luqman et al., 2024; Zhang et al., 2025). The genomic similarity between aMPV isolates within the same subtype is high for an RNA virus, with less than 5% sequence divergence observed among their genomes (Alvarez Narvaez et al., 2025a). Since only a small number of single nucleotide polymorphisms (SNPs) distinguish most isolates, WGS is essential to meaningfully differentiate outbreak viruses and determine epidemiologic relationships. Direct sequencing of clinical or field samples without prior viral genome enrichment often yields data dominated by host-derived reads, with only a small fraction aligning to the viral genome (Alvarez Narvaez et al., 2023). Consequently, the resulting dataset may lack sufficient viral reads to achieve complete genome coverage. Several approaches are currently used for viral RNA genome enrichment (Alvarez Narvaez et al., 2023). These include virion purification prior to RNA extraction (Dent et al., 2015; James et al., 2016), the utilization of double stranded RNA (dsRNA) extraction kits (Gaafar et al., 2020), host ribosomal RNA depletion (Parris et al., 2022), sequence-independent single-primer amplification (SISPA) of the genome (Chrzastek et al., 2017), bait hybridization, or tiled amplicon enrichment (Koskela Von Sydow et al., 2023). Recently, our group successfully retrieved complete aMPV-A genomes from respiratory swab pools using a combination of host ribosomal RNA depletion followed by SISPA for viral genome enrichment (Goraichuk et al., 2024c). Herein, we describe our efforts to develop and test an amplicon-based tiled PCR scheme that improves the sequence sensitivity and enriches for aMPV-B genomes. This new approach is compatible with both short- and long-read sequencing technologies and has proven to be more efficient than the currently used enrichment method combining host RNA depletion and SISPA for aMPV-B genome recovery.

## 2 Materials and methods

### 2.1 Sample collection

Ten chicken oropharyngeal swabs obtained from chicken flocks in the state of Georgia, U.S. were tested aMPV-B positive by the Georgia Poultry Laboratory Network (GPLN) and were submitted to the U.S. National Poultry Research Center (USNPRC) for aMPV whole genome sequencing and analysis.

### 2.2 aMPV-B RT-qPCR

Total RNA was extracted using the MagMAX™-96 Viral RNA Isolation Kit (Applied Biosystems, US), following the manufacturer’s protocol. RT-qPCRs were carried out using a modified version of the Etteradossi scheme and AgPath-ID™ One-Step RT-PCR Reagents (Applied Biosystems) in a QuantStudio5 thermocycler (Applied Biosystems), as previously described (Goraichuk et al., 2024c). Four μL of RNA were added to a 21 μL of master mix consisting of 12.5 μL of 2X RT-PCR buffer, 1 μL of AmPV-B+ primer (20 μM stock), 1 μL of AmPV-B- primer (20 μM stock), 0.5 μL of AmPV-B probe (6 μM stock), 1 μL of 25X enzyme and 5 μL of molecular grade water for a final reaction volume of 25 μL per reaction. PCR reactions were performed in triplicate. The thermocycler conditions were 10 min. at 45 °C of reverse transcription, 10 min. at 95 °C for polymerase activation, and 40 cycles of amplification with 15 s at 95 °C of denaturation, followed by 60 s of oligonucleotide hybridization and elongation at 60 °C. Ct (cycle threshold) values were estimated automatically by QuantStudio™ Design and Analysis Software (v1.5.3, Applied Biosystems). Ct cut off was set at 40 cycles. Ct values below 37 were considered positive while Ct values of 37–40 and greater were reported as suspect.

### 2.3 Depletion of host and bacterial RNA and sequence-independent, single-primer amplification

Total RNA was also extracted using the MagMAX™-96 Viral RNA Isolation. Host rRNA (chicken) and RNA from the most common bacterial pathogens were depleted using our previously published protocol (Goraichuk et al., 2024a; Goraichuk et al., 2024b). Briefly, total RNA was first hybridized with custom ssDNA probes targeting chicken 18S, 28S, and mitochondrial rRNA and bacterial 16S and 23S RNA. Following hybridization, RNase H (New England Biolabs) was used to selectively degrade rRNA molecules bound by the probes. Residual unbound ssDNA probes were then digested using TURBO DNase (TURBO DNA-*free*™ Kit, Invitrogen). The depleted RNA was subsequently purified using 2.2X RNAClean XP beads (Beckman Coulter) according to the manufacturer’s instructions. aMPV cDNA was generated using our sequence-independent, single-primer amplification (SISPA) protocol (Chrzastek et al., 2017). Briefly, viral RNA was first reverse-transcribed into cDNA using SuperScript IV reverse transcriptase (ThermoFisher Scientific) and a degenerate primer (R8N). Following first-strand synthesis, second-strand cDNA synthesis was performed using Klenow polymerase (New England Biolabs). The resulting double-stranded cDNA was purified with X1.8 AMPure XP beads (Beckman Coulter) and then amplified via PCR using the Phusion HiFi DNA polymerase (New England Biolabs) and the RN primer. A final purification step with X1.8 AMPure XP beads was performed to obtain clean amplicons suitable for sequencing.

### 2.4 cDNA synthesis and amplicon-based tiled PCR strategy

RNA was extracted with RNAzol-RT (Molecular Research Center) combined with the Monarch Spin RNA Cleanup Kit (New England Biolabs). Total RNA was first reverse-transcribed into cDNA using the LunaScript^®^ RT SuperMix Kit (New England Biolabs). Five sets of primers were used to amplify the complete length of the aMPV-B genome (**Table 1**) in individual PCR reactions using the Q5^®^ High-Fidelity 2X Master Mix (New England Biolabs). A 45 μL master mix that consisted of 2.5 μL of forward oligo (10 μM stock), 2.5 μL of reverse oligo (10 μM stock), 25 μL of 2X master mix and 15 μL of water was combined with 5 μL of cDNA. Reactions were run in a MiniAmpPlus Thermal Cycler (Thermo Fisher Scientific) thermocycler under the following conditions: an initial denaturation at 98 °C for 24 s followed by 35 cycles of denaturation at 98 °C for 10 seconds, annealing at 62 °C for 30 s, and extension at 72 °C for 2 min. A final extension was performed at 72 °C for 2 min., followed by a hold at 4 °C. Amplicons were visualized by 1% agarose in TAE (Tris-Acetate-EDTA buffer) gel electrophoresis and purified using X1.8 AMPure XP beads. An estimate of the individual amplicon concentrations for each sample was obtained using a NanoDrop spectrophotometer (Thermo Fisher Scientific) and normalized to 20 ng/μL. 5 μL from each of the five normalized PCR products per sample were pooled, and the resulting 25 μL mixture was used for NGS library preparation.

**Table 1:** Amplicon-based tiled PCR scheme for the enrichment of aMPV-B genomes.

Set	Oligo	Sequence 5’- 3’	Amplicon size (bp)
1	AMPV_B Adapter/Leader 1 F	ACGAGAAAAAAACGCATTCAAGTCAC	3,075
	AMPV_B 3075 R	TACCAACCCGTTCTGAGCAC	
2	AMPV_B 2843 F	TGGAACCACCAGAGGACTAGGTAT	3,751
	AMPV_B 6594 R	GTCTGAATGTACCGGAGGAG	
3	AMPV_B 6197 F	GTGGACACTTATTGGGCGGA	2,532
	AMPV_B 8729 R	GCAGAAGCTGATACCTGCTAAC	
4	AMPV_B 6412 F	GCTCTTTGTGAATCAGGACC	3,671
	AMPV_B 10083 R	ACTGTGTCTGACTCACTGCC	
5	AMPV_B 9997 F	AGCAGGTAGTCAGATATCTAATGAAT	3,157
	AMPV_B Trailer 13514 R	ACGGCAAAAAAACCGTATTCAATAC	

Full genome amplification of aMPV_B was further simplified by using only two primers from the previous set by adjusting reaction conditions. Total RNA was obtained as above and reverse transcribed using Maxima H Minus RT (ThermoFisher). Briefly, 1μg RNA was mixed with 1μl of dNTPs and 2 μL of AMPV_B Trailer 13514 R (**Table 1**). Reactions were heated at 65 °C for 5 min. and chilled on ice for 5 min. Next, 4 μL of 5X RT buffer, 0.5 μL ThermoScientific™ RiboLock RNase Inhibitor and 50 Units of Maxima H Minus RT were added to the mix that was subsequently incubated at 50 °C for 90 min. The resulting cDNA was diluted 1:10 in nuclease-free water, and 10 μL were included in each 50 μL PCR reaction together with 25μL of LongAmp™ *Taq* 2X Master Mix (New England Biolabs), 2 μl of forward primer (10 µM stock AMPV_B Adapter/Leader 1 F), 2 μL of reverse primer (10 µM stock AMPV_B Trailer 13514 R), and 11μL nuclease-free water. Reactions were performed as above with an initial denaturation step at 94 °C for 60 s, followed by 30 cycles of denaturation at 94 °C for 30 s, annealing 58 °C for 35 s, and extension at 65 °C for 12 min. A final extension step was performed at 65 °C for 120 min. and hold was set at 4 °C. The entire 50 μl PCR product was separated on a 0.8% agarose gel with a High Molecular Weight Ladder (Invitrogen). Bands corresponding to the aMPV B genome size at 13 Kbp were purified with Monarch^®^ DNA Gel Extraction Kit (New England Biolabs) per the manufacturers recommendations.

### 2.5 Illumina short-read sequencing and bioinformatic analysis

Genomic libraries were prepared using the Illumina DNA Prep (formerly Nextera Flex) Kit (Illumina) along with the IDT for Illumina DNA/RNA UD Indexes Set A (Illumina). Sequencing was carried out on an Illumina MiSeq platform using a MiSeq v2 (250 cycle). Raw read processing was performed at the USNPRC instance of Galaxy bioinformatic platform (The Galaxy Community, 2024). Raw reads were quality-filtered (Phred score > 30) and trimmed using Trimmomatic (Bolger et al., 2014) v0.39+galaxy2. Read quality was assessed with FastQC v0.74. High-quality reads were then aligned to the aMPV-B reference genome Hungary/657/4 (NCBI accession no. MN729604.1) using BWA v7.17.2 (Li, 2013). The resulting BAM alignment files were used to estimate the proportion of mapping reads and the average genome coverage and sequencing depth using SAMtools v1.15.1 (Li et al., 2009). Reference-based assemblies were visualized in Geneious Prime v2025.0.3 (https://www.geneious.com). The Illumina raw reads generated in this study have been deposited in the National Center for Biotechnology Information (NCBI) Sequence Read Archive (SRA) under bioproject PRJNA1387543 (**Supplementary Tables 2**, **3**).

### 2.6 Oxford Nanopore Technology long-read sequencing and bioinformatic analysis

Oxford Nanopore Technologies (ONT) long-read sequencing libraries were prepared using the Native Barcoding Kit 24 V14 (ONT) and run on a SpotON R10.4.1 flow cell (ONT). Raw read processing and subsequent analyses were carried out at the USNPRC Galaxy bioinformatic platform. Raw reads were quality filtered using Filtlong v0.2.1 (https://github.com/rrwick/Filtlong) with a threshold Q value >7, and trimmed with Porechop v0.2.4 (Bonenfant et al., 2023). *De novo* assemblies were performed using Canu 2.2 (Koren et al., 2017), and 13Kb as expected genome size. Reference based assemblies were obtained with minimap2 v2.26 (Li, 2016) and the aMPV-B reference genome Hungary/657/4. Genome coverage and sequencing depth were estimated using SAMtools v1.15.1 from the bam files produced by after minimap2. Multiple sequence alignments were performed using Clustal Omega 1.2.2 (Sievers et al., 2014) in Geneious Prime v2025.0.3. The ONT raw reads generated in this study have been deposited in the NCBI SRA under bioproject PRJNA1387543 (**Supplementary Tables 4**, **5**).

### 2.7 Statistical analysis

Differences in the number of aMPV-B-mapping reads, genomes coverage and sequencing depth between the current genome enrichment strategy (host RNA depletion followed by SISPA) and the tiled PCR scheme were assessed using the Wilcoxon matched-pairs signed-rank test, as the data were paired and did not follow a normal distribution (as determined by the Shapiro-Wilk test). A two-tailed *p* < 0.05 was considered statistically significant. Statistical analyses were performed using GraphPad Prism v10.3.1.

## 3 Results

### 3.1 Development of an amplicon-based tiled PCR scheme for aMPV-B WGS

The amplicon-based tiled PCR scheme (aMPVB-ATP) was designed based on the aMPV-B reference genome Hungary/657/4 (NCBI accession #MN729604) and consist of five primer sets that produce five amplicons between 3,075 and 3,751 bp in length and a minimum overlap of 83 bp, covering the complete aMPV-B genome. Set 1 covers the area of the genome between the leader sequence (site 1bp) to site 3,075 bp, while set 2 amplifies from site 2,843bp to 6,594 bp. Sets 3 and 4 enrich areas between sites 6,197 bp and 8,729 bp, and sites 6,412 bp and 10,083 bp respectively, and set 5 covers the portion of the genome from site 9,997 to the end of the trailer sequence (site 13,514 bp, **Table 1**). The primer sequences were mapped to the 64 aMPV-B genomes used in our previous study (Álvarez-Narváez et al., 2025b) (accession numbers in **Supplementary Table 1**) for in silico validation (**Supplementary Figure 1**). Except for primer AMPV_B 2843 F, which showed two nucleotide mismatches with 26 out of the 50 U.S. genomes, and primer AMPV_B 6594 R, which showed one nucleotide mismatch with U.S. genomes 11283951, 11297877, 11297876, and NC23734, all remaining primers aligned to the American genomes with 100% identity and exhibited a maximum of three mismatches with a limited number of non-American genomes. Overall, these results support the robustness of the primer design and suggest that the aMPVB-ATP scheme is suitable not only for currently circulating U.S. aMPV-B strains but also for a broad range of genetically diverse international aMPV-B isolates, with a low likelihood of amplification failure due to primer–template mismatches.

**Figure 1:**
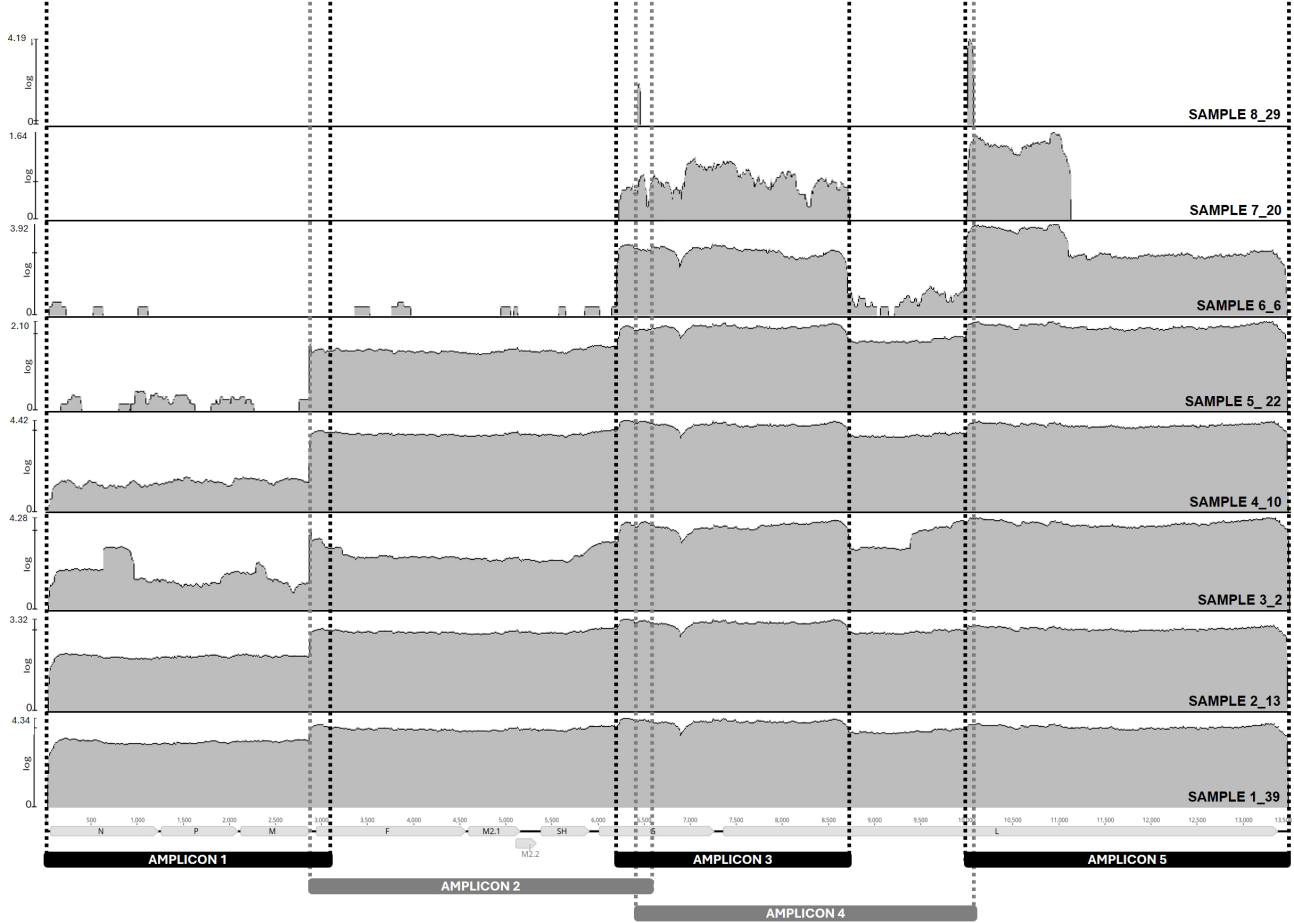
Genome coverage after aMPV-B genome enrichment using the new amplicon-based tiled PCR scheme and Illumina short-read sequencing. Annotated aMPV-B reference genome Hungary/657/4 (NCBI accession #MN729604) was used to map the reads from samples 1_39, 2_13, 3_2, 4_10, 5_22,6_6, 7_20 and 8_29. Samples 9_28 and 10_35 were not included in the analysis because no aMPV-B-mapping reads were obtained after NGS. Sequencing depth (Log_10_ X) is represented in grey (y-axis) for the entire length the genome (x-axis). The area amplified by each primer set is delimited by dotted lines. Reference guided assembly and visualization was performed in Geneious Prime (Geneious Prime 2025.1.1, https://www.geneious.com), and the figure was manually edited in PowerPoint version 2025.

The aMPVB-ATP scheme was tested in ten aMPV-B positive oropharyngeal samples obtained during the 2024 U.S. outbreak with Ct values ranging between 14 and 33 (**Supplementary Table 2**). Samples with Ct values <18 showed all the expected bands when visualized in a 1% agarose gel, while samples with Ct values between 18 and 24 showed some of the bands and samples with Ct values above 28 showed no bands (**Supplementary Figure 2**). Illumina short-read sequencing of the pooled amplicons for the 10 samples yielded 9,664,880 raw reads of which 6,120,358 passed quality filtering. For samples with Ct values ≤ 22, an average of 78% of filtered reads per isolate (586,082 ± 98,589, mean ± SEM) were found to be aMPV-B-mapping reads (**Supplementary Table 2**). The proportion of aMPV-B-mapping reads in samples presenting Ct > 22, dramatically decreased with the increase in Ct values. The samples with Ct values of 25, 28 and 31 yielded 13% (42,164 reads), 1.6% (1,774 reads) and 0.01% (100 reads) of aMPV-B-mapping reads respectively, and no aMPV-B-mapping reads were recovered from the two samples with Ct values >31 (**Table 2**). Consequently, complete genomes (>99% coverage) supported by good sequencing depth (>2,300X) were only recovered from samples presenting Ct values ≤ 22 (**Supplementary Table 2**, **Figure 1**). As observed for the number of reads, the % genome coverage and sequencing depth also decreased with increasing Ct values. Less than 62% of the genome with an average sequencing depth of 178X was recovered from the sample with a Ct value of 25 and only 27% of the genome was found in the sample presenting a Ct value of 28 (7X average sequencing depth). Additionally, in samples with good sequencing depth, a slight decrease in number of reads was observed when travelling from the 5’-end to the 3’-end of the genome (**Table 2**, **Figure 1**).

**Table 2:** Summary of the performance of the amplicon-based tiled PCR scheme in samples presenting different Ct values.

Sample ID	Ct-value cDNA	Amplicon 1		Amplicon 2		Amplicon 3		Amplicon 4		Amplicon 5		Total % aMPV filtered reads
		Visible band	% aMPV-B mapping reads	Visible band	% aMPV-B mapping reads	Visible band	% aMPV-B mapping reads	Visible band	% aMPV-B mapping reads	Visible band	% aMPV-B mapping reads	
1_39	14.3	Yes	4.48	Yes	19.60	Yes	15.17	Yes	18.97	Yes	23.92	82.14
2_13	18.1	Yes	1.21	Yes	18.33	Yes	16.10	Yes	20.16	Yes	23.23	79.04
3_2	18.1	No	1.05	Yes	3.54	Yes	11.35	Yes	16.06	Yes	41.21	73.22
4_10	22.7	No	0.35	Yes	15.62	Yes	13.57	Yes	16.33	Yes	36.09	81.97
5_22	22.4	No	0.23	Yes	4.84	Yes	13.65	Yes	16.17	Yes	41.11	76.00
6_6	25.2	No	0.00	No	0.26	Yes	1.41	Yes	1.28	Yes	10.00	12.96
7_20	29.7	No	0.02	No	0.17	No	0.22	No	1.16	No	0.00	1.57
8_29	31.3	No	0.00	No	0.00	No	0.00	No	0.01	No	0.00	0.01
9_28	32.5	No	0.00	No	0.00	No	0.00	No	0.00	No	0.00	0.00
10_35	33.4	No	0.00	No	0.00	No	0.00	No	0.00	No	0.00	0.00

Performance is measured as the proportion of aMPV-B-mapping reads for each primer set.

### 3.2 The amplicon-based tiled PCR scheme is superior to the combination of host RNA depletion and SISPA for the enrichment of the aMPV-B genomes from respiratory samples

**Figure 2:**
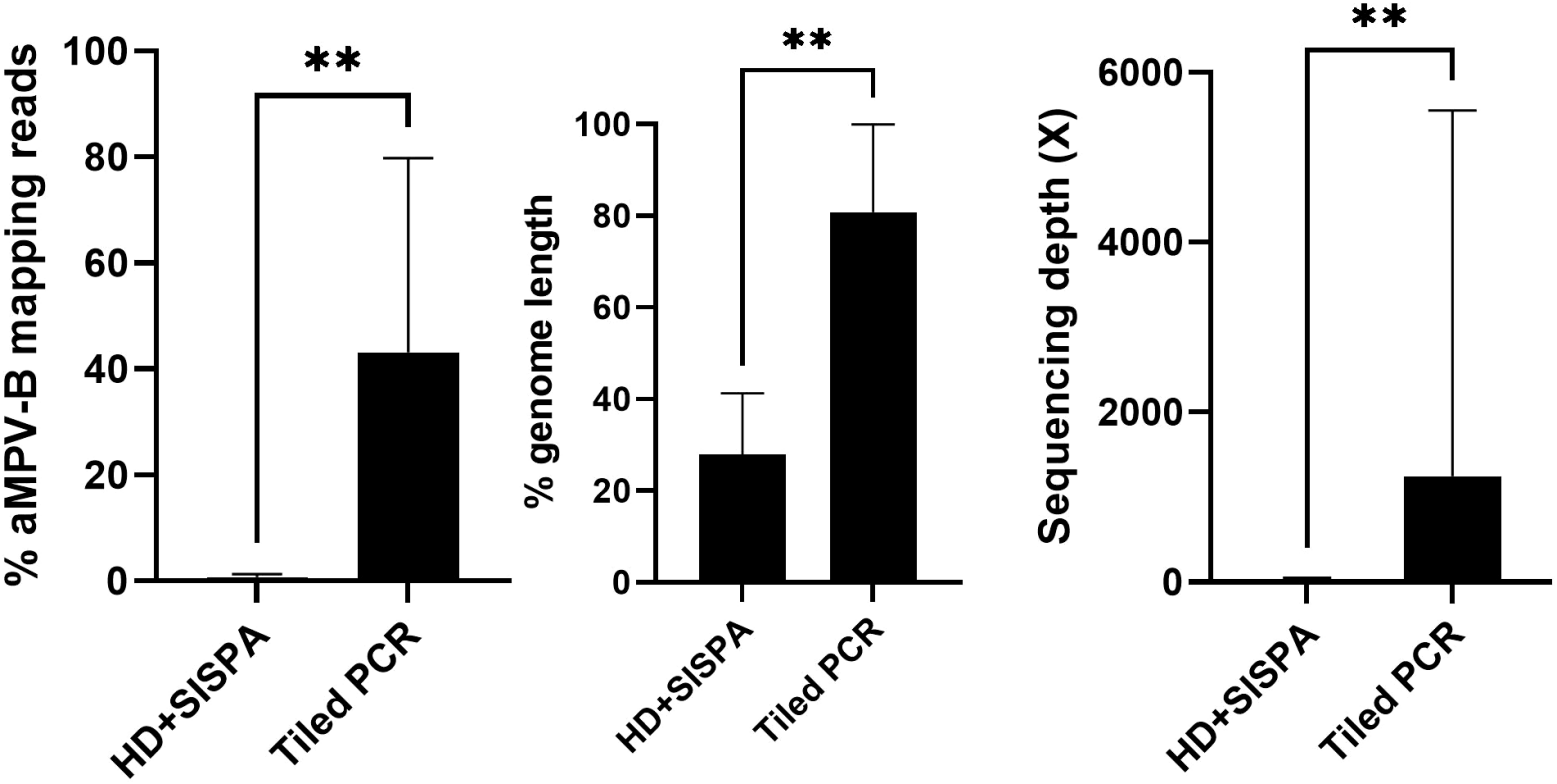
Bar plots comparing **(A)** the proportion of aMPV-B-mapping reads, **(B)** percentage of genome covered and **(C)** sequencing depth for each bp of the genome obtained with the two genome enrichment strategies: host RNA depletion combined with SISPA (HD+SISPA) or the amplicon-based tiled PCR approach (Tiled PCR). Data are presented as median with interquartile range. A *p* < 0.05 was considered statistically significant. GraphPad Prism v10.3.1 was used to perform statistical analyses and to produce the graphs. Two asterisks represent a highly significant difference with an estimated p <0.01.

The same ten aMPV-B positive oropharyngeal samples were subjected to host/bacterial RNA depletion and SISPA (HD+SISPA), to compare the performance of the current enrichment strategy with the new aMPVB-ATP scheme. Illumina short-read sequencing for the 10 samples yielded a total of 11,454,005 raw reads of which 10,229,070 passing quality filtering (1,022,907 ± 156,878 reads [mean ± SEM] per isolate; **Supplementary Table 3**). As observed with the tiled PCR approach, the proportion of aMPV-mapping reads decreased with the increase in Ct values. For most of the isolates, the proportion of filtered reads that mapped with the aMPV-B reference genome never exceeded 1%. Only sample 1_39 with the lowest Ct value (Ct = 16) showed a proportion of mapping reads close to 6% (**Supplementary Table 3**). When compared to the amplicon-based tiled PCR approach, HD+SISPA yielded significantly (*n* = 10, median difference 42.85%, *p* = 0.0078) less proportion of aMPV reads. Similarly, the HD+SISPA enrichment allowed for the recovery of significantly less complete genomes (*n* = 10, median difference 13.94%, *p* = 0.0078) and with significantly less sequencing depth (*n* = 10, median difference 1,246X, *p* = 0.0078) than the tiled PCR strategy (**Figure 2**), evidencing that the latter is a more efficient method for aMPV-B genome sequencing.

### 3.3 The aMPV-B amplicon-based tiled PCR scheme can be combined with short- and long- read NGS

**Figure 3:**
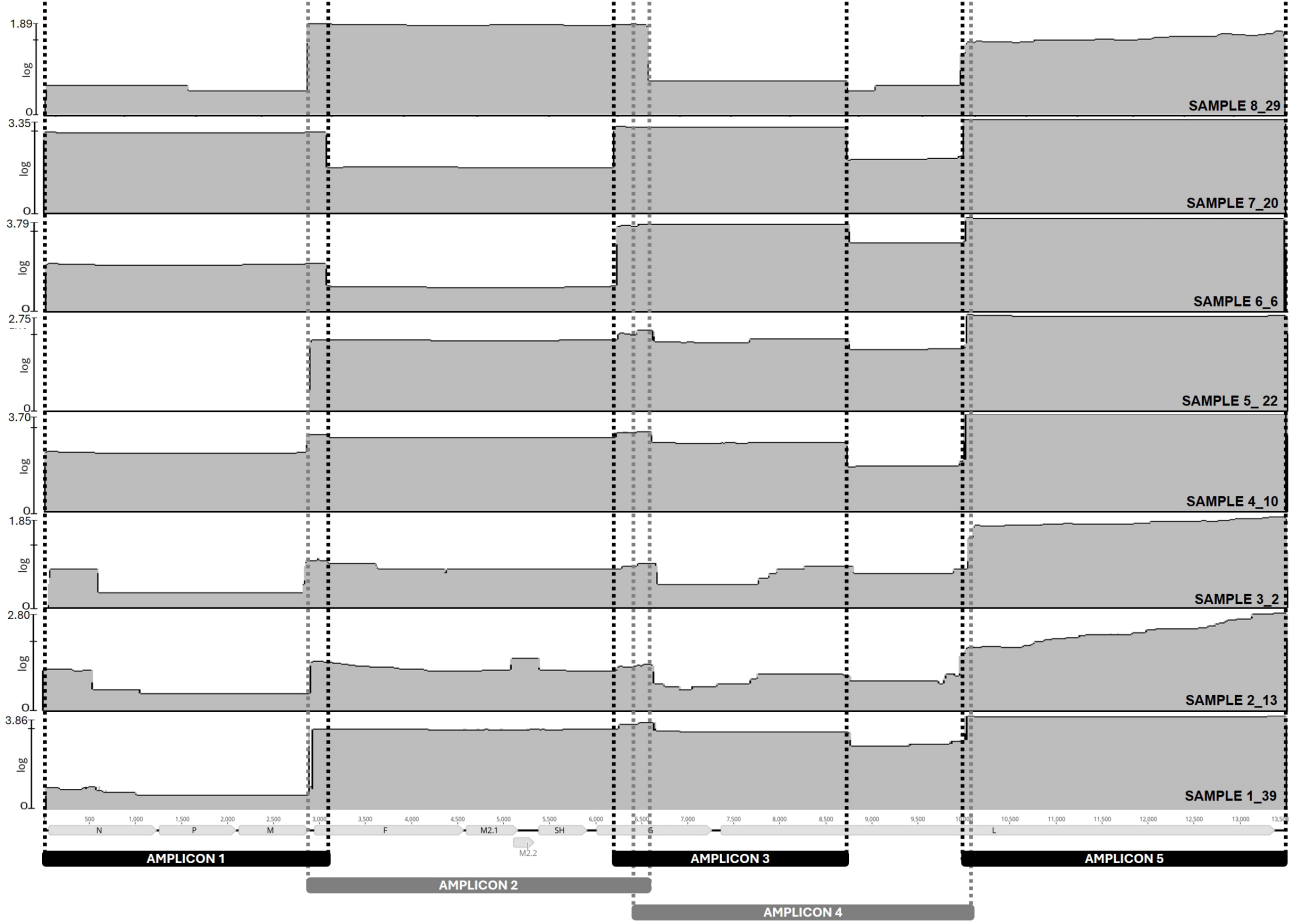
Genome coverage after aMPV-B genome enrichment using the new amplicon-based tiled PCR scheme and ONT long-read sequencing. Annotated aMPV-B reference genome Hungary/657/4 (NCBI accession #MN729604) was used to map the reads from samples 1_39, 2_13, 3_2, 4_10, 5_22, 6_6, 7_20 and 8_29. Samples 9_28 and 10_35 were not included in the analysis because no aMPV-B-mapping reads were obtained after NGS. Sequencing depth (Log_10_ X) is represented in grey (y-axis) for the entire length the genome (x-axis). The area amplified by each primer set is delimited by dotted lines. Reference guided assembly and visualization was performed in Geneious Prime (Geneious Prime 2025.1.1, https://www.geneious.com), and the figure was manually edited in PowerPoint version 20.

The amplicons obtained using the new aMPVB-ATP scheme were also sequenced using ONT long-read sequencing technology (**Supplementary Table 4**). A total of 154,589 raw reads corresponding to 216,222,839 bp were obtained for the ten samples (average 21,622,284 ± 4,642,996 bp [mean ± SEM] per sample). Around 92% of the total bp were recovered after quality filtering and trimming (average 19,906,883 ± 4,537,392 bp [mean ± SEM]). As observed with the short-read data, samples with lower Ct values (Ct < 24) presented a higher proportion of aMPV-B mapping reads, and those reads were also longer (2,336 bp average). This resulted in a higher proportion of the data generated with these samples belonging to aMPV-B genomes (average of 89% of bp per sample). On the contrary, the proportion aMPV-B mapping reads from the samples with Ct values > 24 was low, never reaching 10%. The length of the reads was also shorter (on average <1,293 bp) and therefore the proportion of aMPV-B related data in these samples was also low (average of 14% of bp per sample). Nevertheless, complete genomes (% of genome covered > 99%) were obtained for all samples except for sample S5-22 (**Figure 3**). The main difference between the low and high Ct samples is that the later presented lower sequencing depth (**Supplementary Table 4**). There was an overall decrease in the reads that mapped with the 5’-end to the genome compared to the 3’-end (**Figure 3**), matching with what previously observed when mapping the Illumina short reads. Sample 5-22, the only sample for which a complete genome assembly was not obtained, did not have any reads mapping the region of the genome covered by the amplicon 1 (**Figure 3**), indicating that a problem in the PCR amplification step, and not the actual sequencing, most probably caused the resulting incomplete genome. For the samples that yielded complete genomes after aMPVB-ATP enrichment and Illumina sequencing (those with Ct values ≤ 22), pairwise alignments between assemblies generated with aMPVB-ATP combined with Illumina and aMPVB-ATP combined with ONT were performed to determine the sequence similarity. The alignments showed a minimum nucleotide sequence similarity of >99.7%, indicating that the assemblies were very similar and only diverged at a few nucleotide positions (**Supplementary Table 4**).

A long PCR scheme using primers AMPV_B Adapter/Leader 1 F and AMPV_B Trailer 13514 R (**Table 1**) was tried in all samples to amplify the complete aMPV genome in one single reaction. Unfortunately, we only obtained amplicons for samples S1-39, S2-13, S3-2, S4–10 and S6-6, all with Ct ≤ 24. ONT long-read sequencing of the five samples yielded similar results to the ones obtained using the tiled PCR approach but with a less proportion (<77.47%) and shorter (908 ± 319 bp [mean ± SEM]) aMPV-B-mapping reads (**Supplementary Table 5**). That in turn translated into a slightly lower proportion of the genome being covered and with a lower depth (**Supplementary Table 5**).

## 4 Discussion

Genomic data generated during a viral outbreak provides critical insights that support both immediate response and long-term control. Specifically, genomic information facilitates the detection of mutations associated with enhanced virulence (Chakraborty et al., 2025), antigenicity (Sun et al., 2022), or diagnostic escape (Ko et al., 2022), thereby ensuring that molecular assays remain reliable and that vaccines are appropriately matched to circulating strains. Additionally, genomic comparative analyses allow the determination of viral transmission dynamics, revealing whether outbreaks result from a single introduction or multiple independent events (Alvarez Narvaez et al., 2025a).

While the current aMPV outbreak in the US has spread to 32 states, the genomic data available is still limited. One reason for the lack of data may be the difficulty in obtaining complete aMPV genomes directly from clinical samples. This hypothesis is supported by the observation that, in this study, only two out of ten complete genomes were recovered without the use of the amplicon-based tiled PCR scheme, and this was only possible after viral genome enrichment through host RNA depletion and SISPA, and from samples with the lowest Ct values. One approach that virologists use in order to improve their chances to recover complete viral genomes is the propagation of the pathogen to increase its titers before nucleotide extraction and NGS. However, unlike many avian viruses, aMPV does not propagate efficiently in embryonated chicken eggs and has been found to replicate in low titers in primary avian cells and tracheal organ cultures (TOCs) (Suarez et al., 2020). Documented efforts for isolation of aMPV subtypes A and B during this outbreak have been so far unsuccessful in Vero cells, DF-1 cells and 9-day-old embryonated chicken eggs (ECEs) inoculated via allantoic cavity route (Zhang et al., 2025). A few clinical isolates have been recovered in either primary chicken embryo fibroblast (CEF) and lung (CEL) cells and adapted to grow in the Vero cell line following primary isolation (Zhang et al., 2025). However, forcing the adaptation of a clinical isolate to grow in a cell line may promote genetic changes that alter its original genomic characteristics and pathogenicity (Funnell et al., 2021). The amplicon-based tiled PCR scheme allows sequencing directly from clinical samples, in this case oropharyngeal swabs. Compared with other molecular approaches for viral genome enrichment, such as host RNA depletion combined with SISPA, aMPVB-ATP scheme yielded more complete genomes, including from samples with higher Ct values. The combination of host RNA depletion and SISPA has been successfully applied for the recovery of complete genomes of RNA viruses that intrinsically replicate well in cell culture (Alvarez Narvaez et al., 2023; Álvarez-Narváez et al., 2025a). However, in the case of aMPV, we believe that for clinical samples with low viral titers, the host RNA depletion step further reduces the already limited viral genomic RNA which results in inefficient SISPA and therefore a less suitable approach.

The aMPVB-ATP genome enrichment method proposed here can also be combined with ONT sequencing without the need for enzymatic depletion steps, making it a more affordable option for small or resource-limited laboratories. In fact, the application of ONT sequencing after enrichment appeared to provide a higher number of complete genomes compared to when Illumina was used. This can be due to differences in sequencing chemistry. While ONT library prep preserves and sequences intact amplicons as single molecules (Wang et al., 2021), Illumina requires fragmentation (Illumina, 2014), which can disproportionately affect genome recovery from low-abundance or unevenly amplified regions. One of the advantages of using ONT is its ability to generate long reads ranging from 10–100 kb when run in standard long-read mode (Wang et al., 2021). Based on this, we attempted to amplify the complete aMPV-B genome in a single PCR reaction. However, this approach was only successful for samples with lower Ct values, likely because high-Ct (low-titer) samples did not provide sufficient template for full-length amplification. Nevertheless, complete or nearly complete (>95% length covered) genomes were recovered for all samples that could be PCR amplified.

The assemblies generated from Illumina and ONT data showed high pairwise sequence identity (< 41 nucleotide differences) but not complete agreement, most likely reflecting the higher per-base error rate typically associated with ONT sequencing, particularly when compared to Illumina datasets with high sequencing depth (>2,300× coverage in this study). The genetic diversity observed among aMPV-B isolates recovered during the 2024 U.S. outbreak sometimes did not exceed 29 nucleotides (Álvarez-Narváez et al., 2025b), indicating that the number of differences introduced between sequencing platforms is comparable to the number of nucleotides separating some outbreak isolates. Hence, while the aMPVB-ATP enrichment approach combined with ONT sequencing enables recovery of complete genomes from samples with higher Ct values, ONT-only assemblies may not be optimal for high-resolution SNP-based analyses. If accurate SNP identification is required, increasing the Illumina sequencing depth after aMPVB-ATP enrichment and/or combining the Illumina reads with the ONT data to generate hybrid assemblies could improve consensus accuracy (Wick et al., 2017).

The main limitation of this study is that the aMPVB-ATP method was tested in only ten samples, all collected from chicken flocks in Georgia (US) during the 2023–24 outbreak. Therefore, its performance and robustness across a larger and more diverse set of field samples remains to be validated. Still, in our in silico validation most primers demonstrated complete (100%) identity across U.S. genomes and only limited mismatches among a small subset of international isolates. Importantly, the observed mismatches were limited in number and not predicted to substantially impair amplification efficiency, particularly given the use of high-fidelity polymerases and relatively long amplicon targets (Letowski et al., 2004). Nonetheless, because amplicon-based approaches are inherently dependent on primer-template complementarity, continued genomic surveillance is essential. The emergence of additional genetic diversity within primer binding regions could reduce amplification efficiency or lead to dropout of specific amplicons. Therefore, to ensure comprehensive variant detection moving forward, periodic re-evaluation of primer binding sites against newly generated aMPV-B sequences is recommended.

aMPVB-ATP has proven to be a novel and effective genome enrichment approach that, when combined with NGS, enables recovery of complete aMPV-B genomes with high sequencing depth. Its versatility is further underscored by its compatibility with both short-read (Illumina) and long-read (ONT) sequencing technologies, allowing for straightforward scalability. Furthermore, given the concurrent circulation of aMPV subtypes A and B during the 2023–24 U.S. outbreak, an important consideration is whether the amplicon-based tiled PCR strategy described here for subtype B could be extended to aMPV-A. Because the tiled PCR approach relies on subtype-matched primer binding across the genome, direct application of the current aMPVB-ATP primers to aMPV-A would likely result in suboptimal amplification efficiency due to sequence mismatches. However, the general design framework is readily transferable. Using available U.S. aMPV-A genomic data, including recently characterized outbreak strains (Goraichuk et al., 2024c; Zhang et al., 2025), a subtype A–specific tiled primer scheme could be designed following the same principles applied here. Expanding this strategy to include subtype A would provide a unified genomic surveillance framework capable of supporting real-time molecular epidemiology during multi-subtype outbreaks.

## Data Availability

The datasets presented in this study can be found in online repositories. The names of the repository/repositories and accession number(s) can be found in the article/**Supplementary Material**.
