# Supplementary material for: An amplicon-based tiled PCR scheme for the enrichment of avian metapneumovirus subtype B genomes prior to next generation sequencing
Source: Front Cell Infect Microbiol. 2026 Mar 17;16:1782197. doi: 10.3389/fcimb.2026.1782197 (PMC13036218; doi:10.3389/fcimb.2026.1782197)
Supplement: Supplementary file 1 [file Table1.docx]

Supplementary Material

**Supplementary Table 1. aMPV-B genomes publicly available used in this study**

| **Strain** | **Subtype** | | **NCBI**  **Accession No** | **Country of**  **Isolation** | **Host** |
| --- | --- | --- | --- | --- | --- |
| aMPV-B/BR/1890/E1/19 | | B | OP572408 | Brazil | Grown virus |
| aMPV-B/BR/1891/E2/19 | | B | OP572409 | Brazil | Vero cells |
| B1 | | B | PQ7383333 | China | Chicken |
| WH2022 | | B | OP036743 | China | Vero cells |
| LN16 | | B | MH745147 | China | Chicken |
| LN16-A | | B | PP069785 | China | Chicken |
| VCO3/60616 | | B | AB548428 | France | Turkey |
| 657/4 | | B | MN729604 | Hungary | Turkey |
| 21004/2021 | | B | OM249786 | South Korea | Chicken |
| 21004-PLQ7/2021 | | B | OM249787 | South Korea | Chicken |
| SC1509 | | B | OR461286 | South Korea | Chicken |
| N19-29 | | B | OP924007 | South Korea | Chicken |
| N19-83 | | B | OP924006 | South Korea | Chicken |
| N19-41 | | B | OP924005 | South Korea | Chicken |
| ADRDL-2 | | B | PP273457 | USA | Turkey |
| SEP-RS1 | | B | PQ382890 | USA | Turkey |
| ADRDL-1 | | B | PP273456 | USA | Turkey |
| ADRDL-3 | | B | PP273458 | USA | Turkey |
| ADRDL-4 | | B | PP273459 | USA | Turkey |
| ADRDL-5 | | B | PP273460 | USA | Turkey |
| ADRDL-6 | | B | PP273461 | USA | Chicken |
| NC20487_GA/2024_PrimCellsP3 | | B | PV067043 | USA | Turkey |
| NC23734-GA/2024_PrimCellsP1 | | B | PV067047 | USA | Turkey |
| 11281543 | | B | PX124514 | USA | Chicken |
| 11281545.1 | | B | PX124481 | USA | Chicken |
| 11281545.2 | | B | PX124507 | USA | Chicken |
| 11281620 | | B | PX124506 | USA | Chicken |
| 11282511 | | B | PX124513 | USA | Chicken |
| 11282879 | | B | PX124512 | USA | Chicken |
| 11283261 | | B | PX124511 | USA | Chicken |
| 11283628 | | B | PX124505 | USA | Chicken |
| 11283629 | | B | PX124510 | USA | Chicken |
| 11283951 | | B | PX124515 | USA | Chicken |
| 11283954.1 | | B | PX124504 | USA | Chicken |
| 11283954.2 | | B | PX124501 | USA | Chicken |
| 11283954.3 | | B | PX124480 | USA | Chicken |
| 11283957 | | B | PX124516 | USA | Chicken |
| 11284246 | | B | PX124502 | USA | Chicken |
| 11284269.1 | | B | PX124508 | USA | Chicken |
| 11284269.2 | | B | PX124475 | USA | Chicken |
| 11284396.1 | | B | PX124509 | USA | Chicken |
| 11284396.2 | | B | PX124498 | USA | Chicken |
| 11284397.2 | | B | PX124500 | USA | Chicken |
| 11285434.1 | | B | PX124477 | USA | Chicken |
| 11285434.2 | | B | PX124478 | USA | Chicken |
| 11285625.1 | | B | PX124476 | USA | Chicken |
| 11285625.2 | | B | PX124497 | USA | Chicken |
| 11285649 | | B | PX124496 | USA | Chicken |
| 11285667 | | B | PX124495 | USA | Chicken |
| 11289366 | | B | PX124494 | USA | Chicken |
| 11289371.1 | | B | PX124493 | USA | Chicken |
| 11293896 | | B | PX124492 | USA | Chicken |
| 11295603 | | B | PX124490 | USA | Chicken |
| 11295604 | | B | PX124491 | USA | Chicken |
| 11295731 | | B | PX124489 | USA | Chicken |
| 11295822 | | B | PX124499 | USA | Chicken |
| 11295929.1 | | B | PX124487 | USA | Chicken |
| 11295929.2 | | B | PX124488 | USA | Chicken |
| 11297143.1 | | B | PX124486 | USA | Chicken |
| 11297143.2 | | B | PX124485 | USA | Chicken |
| 11297876 | | B | PX124484 | USA | Chicken |
| 11297877 | | B | PX124483 | USA | Chicken |
| 11306270 | | B | PX124482 | USA | Chicken |
| 11284245.a | | B | PX124479 | USA | Chicken |
| 11284245.d | | B | PX124503 | USA | Chicken |

**Supplementary Table 2. Illumina short-read sequencing results for the amplicon-based tiled PCR enrichment method**

| **Amplicon-based tiled PCR scheme** | | | | | | | | |
| --- | --- | --- | --- | --- | --- | --- | --- | --- |
| **Sample ID** | **Ct-value cDNA** | **Raw reads** | **Filtered reads** | **Filtered reads mapping to AMPV-B** | **% aMPV mapping reads** | **% Genome Covered** | **Sequencing depth (X)** | **NCBI accession No.** |
| **1_39** | 14 | 951,210 | 663,850 | 545,297 | 82.14 | 99.96 | 5232.74 | SRR36474005 |
| **2_13** | 18 | 1,280,416 | 796,990 | 583,526 | 73.22 | 99.97 | 5157.90 | SRR36474004 |
| **3_2** | 18 | 1,836,456 | 1,060,032 | 837,841 | 79.04 | 99.99 | 7694.93 | SRR36474003 |
| **4_10** | 22 | 1,385,346 | 870,898 | 713,846 | 81.97 | 99.99 | 6510.40 | SRR36474002 |
| **5_22** | 22 | 609,226 | 328,834 | 249,898 | 76.00 | 99.26 | 2318.84 | SRR36474001 |
| **6_6** | 25 | 494,376 | 325,338 | 42,164 | 12.96 | 62.34 | 178.05 | SRR36474000 |
| **7_20** | 28 | 206,266 | 113,342 | 1,774 | 1.57 | 27.02 | 6.86 | SRR36473999 |
| **8_29** | 31 | 1,317,514 | 904,472 | 100 | 0.01 | 0.85 | 0.19 | SRR36473998 |
| **9_28** | 32 | 1,010,090 | 678,878 | 0.00 | 0.00 | 0.00 | 0.00 | SRR36473997 |
| **10_35** | 33 | 573,980 | 377,724 | 0.00 | 0.00 | 0.00 | 0.00 | SRR36473996 |

**Supplementary Table 3. Illumina short-read sequencing results for host RNA depletion and SISPA enrichment strategy**

| **Host depletion and SISPA** | | | | | | | | |
| --- | --- | --- | --- | --- | --- | --- | --- | --- |
| **Sample ID** | **Ct-value from sample** | **Raw reads** | **Filtered reads** | **Filtered reads mapping to AMPV** | **% aMPV mapping reads** | **% Genome covered** | **Sequencing depth (X)** | **NCBI accession No.** |
| **1_39** | 16 | 935,354 | 639,238 | 36,798 | 5.76 | 99 | 149 | SRR36491316 |
| **2_13** | 19 | 923,948 | 918,032 | 7,982 | 0.87 | 99 | 129 | SRR36491315 |
| **3_2** | 21 | 1,037,738 | 1,019,228 | 4,932 | 0.48 | 0.99 | 71 | SRR36491314 |
| **4_10** | 23 | 1,672,560 | 1,654,248 | 8 | 0.00 | 8 | 0.11 | SRR36491313 |
| **5_22** | 24 | 2,067,186 | 2,056,910 | 335 | 0.02 | 63 | 5 | SRR36491312 |
| **6_6** | 28 | 1,261,916 | 1,256,266 | 14 | 0.00 | 8 | 0.15 | SRR36491311 |
| **7_20** | 31 | 824,388 | 818,880 | 0 | 0.00 | 0 | 0 | SRR36491310 |
| **8_29** | 33 | 1,033,308 | 704,832 | 0 | 0.00 | 0 | 0 | SRR36491309 |
| **9_28** | 32 | 880,538 | 599,230 | 0 | 0.00 | 0 | 0 | SRR36491308 |
| **10_35** | 32 | 816,828 | 562,206 | 0 | 0 | 0 | 0 | SRR36491307 |

**Supplementary Table 4. ONT long-read sequencing results for the amplicon-based tiled PCR enrichment method**

| **Sample ID** | **1-39** | | **2-13** | | **3-2** | | **4-10** | | **5-22** | |
| --- | --- | --- | --- | --- | --- | --- | --- | --- | --- | --- |
| **No. Raw Reads** | 11,559 | (35,732,304 bp) | 15,098 | (46,239,961 bp) | 13,004 | (31,454,585 bp) | 17,991 | (29,863,408 bp) | 10,747 | (2,603,827 bp) |
| **No. Filtered Reads** | 11,524 | (34,359,375 bp) | 15,046 | (44,460,690 bp) | 12,957 | (29,930,994 bp) | 17,829 | (27,773,192 bp) | 904 | (2,510,254 bp) |
| **No. aMPVB-mapping Reads** | 11,403 | (34,008,723 bp) | 14,907 | (44,384,538 bp) | 9,947 | (28,268,312 bp) | 6,664 | (20,599,625 bp) | 831 | (2,421,876 bp) |
| **Mean Filtered Read Quality** | 14.7 | | 14.6 | | 14.5 | | 14.2 | | 14.4 | |
| **% aMPVB-associated bp** | 98.98 | | 99.83 | | 94.44 | | 74.17 | | 96.48 | |
| **aMPV-B Read Mean length** | 3,008 bp | | 2,863 bp | | 2,841 bp | | 3,091 bp | | 3,105 bp | |
| **% aMPV-B Genome Covered** | 100 | | 100 | | 100 | | 100 | | 78,98 | |
| **Sequencing Depth** | 2497 | | 3252 | | 2044 | | 1497 | | 177 | |
| **% Pairwise identity with Illumina assemblies** | 99.8 (41 mismatches) | | 99.9 (11 mismatches) | | 99.96 (5 mismatches) | | 99.9 (12 mismatches) | | 100 (0 mismatches) | |
| **NCBI Accession No.** | SRR36470604 | | SRR36470603 | | SRR36470597 | | SRR36470596 | | SRR36470595 | |

| **Sample ID** | **6-6** | | **7-20** | | **8-29** | | **9-28** | | **10-35** | |
| --- | --- | --- | --- | --- | --- | --- | --- | --- | --- | --- |
| **No. Raw Reads** | 12,862 | (19,801,910 bp) | 43,004 | (1,9371,344 bp) | 9,756 | (5,991,147 bp) | 4,597 | (2,769,115 bp) | 15,971 | (22,395,238 bp) |
| **No. Filtered Reads** | 12,771 | (18,270,485 bp) | 42,358 | (14,172,160 bp) | 9,671 | (4,843,977 bp) | 4,551 | (2,206,948 bp) | 15,886 | (20,540,757 bp) |
| **Mean Filtered Read Quality** | 13.9 | | 13.6 | | 13.9 | | 13.4 | | 14.0 | |
| **No. aMPVB-mapping Reads** | 4,585 | (13,127,958 bp) | 806 | (2,467,786 bp) | 148 | (452,607 bp) | 99 | (258,006 bp) | 1,285 | (3,951,937 bp) |
| **% aMPVB-associated bp** | 71.85 | | 17.41 | | 9.34 | | 11.69 | | 19.24 | |
| **aMPV-B Read Mean Length** | 2,863 bp | | 3,058 bp | | 3,038 bp | | 2,580 bp | | 3,073 bp | |
| **% aMPV-B Genome Covered** | 100 | | 99.97 | | 99.96 | | 99.89 | | 99,98 | |
| **Sequencing Depth** | 957 | | 177 | | 31 | | 17 | | 283 | |
| **% Pairwise identity with Illumina assemblies** | - | | - | | - | | - | | - | |
| **NCBI Accession No.** | SRR36470594 | | SRR36470593 | | SRR36470592 | | SRR36470591 | | SRR36470590 | |

**Supplementary Table 5. ONT long-read sequencing results for the long PCR enrichment method**

| **Sample ID** | **S1-39** | | **S2-13** | | **S3-2** | | **S4-10** | | **6-6** | |
| --- | --- | --- | --- | --- | --- | --- | --- | --- | --- | --- |
| **No. Raw Reads** | 14,782 | (2,258,162 bp) | 16,305 | (11,793,821 bp) | 5,414 | (2,921,712 bp) | 12,009 | (8,937,605 bp) | 4,870 | (3,251,352 bp) |
| **No. Filtered Reads** | 990 | (2,161,376 bp) | 16,252 | (10,077,676 bp) | 4,186 | (2,214,967 bp) | 11,983 | (7,709,584 bp) | 4,826 | (2,736,357 bp) |
| **No. aMPVB-mapping Reads** | 767 | (2,158,492 bp) | 12,343 | (9,501,029 bp) | 639 | (787,017 bp) | 887 | (758,242 bp) | 742 | (1,003,886 bp) |
| **Mean Filtered Read Quality** | 14.1 | | 14.7 | | 14.6 | | 14.1 | | 14.0 | |
| **% aMPVB-associated bp** | 99.87 | | 94.28 | | 35.53 | | 9.84 | | 36.69 | |
| **aMPV-B Read Mean Length** | 2,183 bp | | 620 bp | | 529 bp | | 643 bp | | 567 bp | |
| **% aMPV-B Genome Covered** | 100 | | 98.16 | | 94.45 | | 99.93 | | 100 | |
| **Sequencing Depth** | 149 | | 665 | | 52 | | 51 | | 63 | |
| **NCBI Accession No.** | SRR36470602 | | SRR36470598 | | SRR36470601 | | SRR36470599 | | SRR36470600 | |

**Supplementary Figure 1. Sequence alignments for 64 aMPV-B genomes to aMPVB-ATP primers. aMPV-B genome Hungary/657/4 (NCBI accession #MN729604), used as a template for primer design, was used as a reference genome. Discrepancies between the genomes and the primers were depicted in different colors, red for A, green for T, blue for C and yellow for G. Alignments were produced and visualized in Geneious Prime (Geneious Prime 2025.1.1, https://www.geneious.com), and the figure was manually edited in PowerPoint version 2025**

**
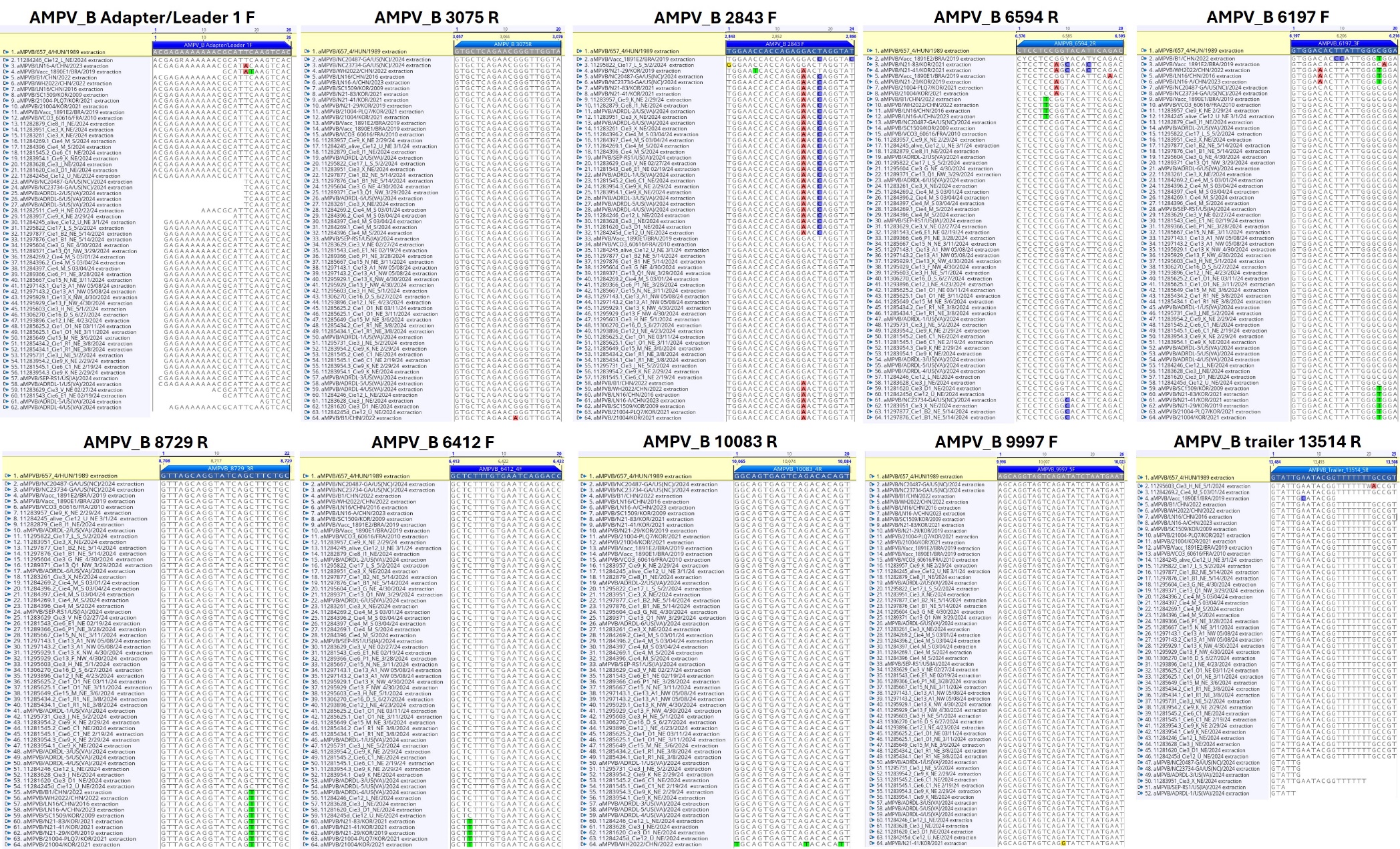
**

**Supplementary Figure 2. 0.8% agarose gel electrophoresis of the aMPVB-ATP amplicons for samples 1_39, 2_13, 3_2, 4_10, 5_22, 6_6, 7_20, 8_29, 9_28, 10_35. Numbers 1 to 5 indicate the primer set used for each PCR reaction as specified in Table 1.**

**
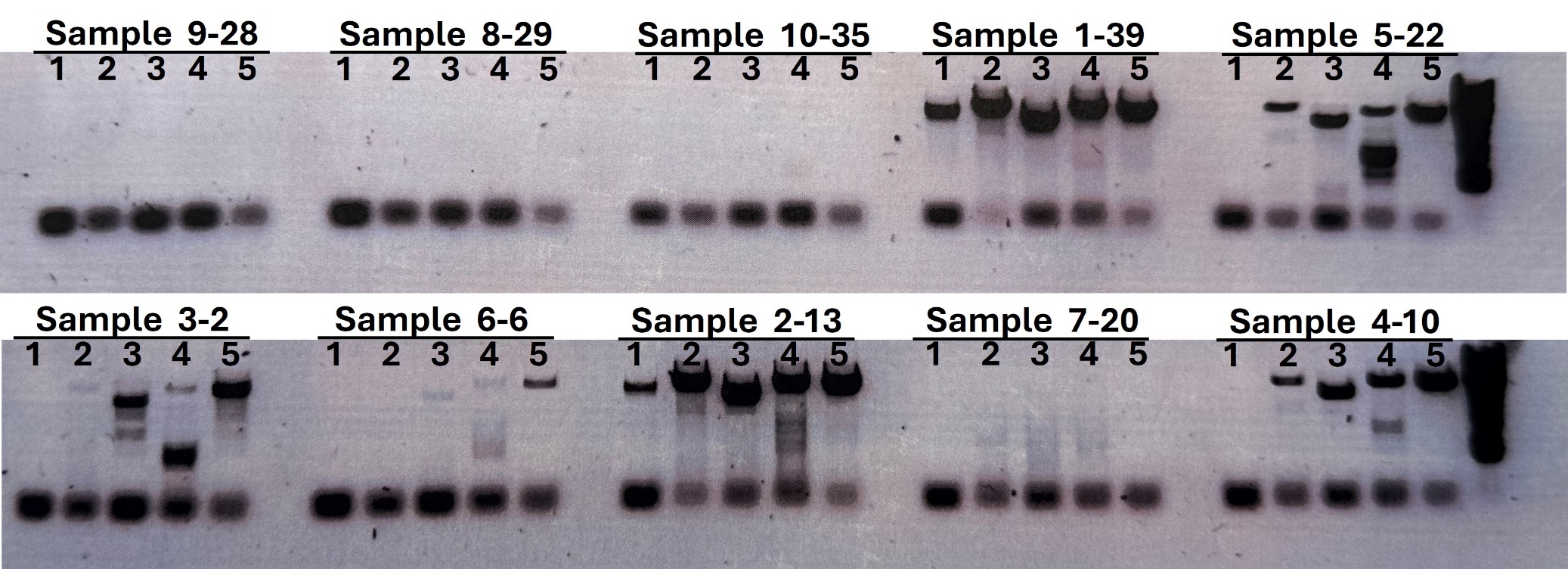
**
